# Prevalence of non‐steroidal anti‐inflammatory drugs (NSAIDs) use in patients with hypertensive crisis

**DOI:** 10.1002/hsr2.483

**Published:** 2022-01-12

**Authors:** Soodeh Jahangiri, Seyed Hamidreza Mousavi, Mohammad Reza Hatamnejad, Maryam Salimi, Hamed Bazrafshan

**Affiliations:** ^1^ Student Research Committee Shiraz University of Medical Sciences Shiraz Iran; ^2^ Al‐Zahra Charity Hospital, Department of Cardiology Medicine Shiraz University of Medical Sciences Shiraz Iran

**Keywords:** adverse effects, hypertension, hypertensive crisis, non‐steroidal anti‐inflammatory drugs

## Abstract

**Backgound:**

One of the known risk factors for hypertensive crisis (HTN‐C) is non‐steroidal anti‐inflammatory drugs (NSAIDs) which their adverse effects can lead to end‐organ damage such as gastrointestinal and cardiovascular issues.

**Aims:**

Data on the correlation between NSAIDs and HTN‐C are limited. In this study, we determined the prevalence of NSAID use among patients with HTN‐C.

**Materials & Methods:**

This cross‐sectional study was conducted among patients primarily diagnosed with HTN‐C referred to Alzahra hospital, Shiraz, Iran from April 2015 to April 2020. Demographic data, as well as information regarding the past medical and drug history and laboratory findings, were gathered retrospectively. The history of NSAID use was also asked specifically. The collected data were analyzed by SPSS and the *P*‐value less than .05 was considered significant.

**Results:**

A total of 257 patients with a mean age of 59.73 were enrolled in the study. Among them 62.6% were female and 137 patients (53.33%) used NSAIDs. Of all the patients 197 (76.7%), 71 (27.6%), and 46 (17.9%) suffered from concomitant hypertension (HTN), diabetes mellitus (DM), and ischemic heart disease (IHD) respectively. A significant relation was found between having each of the comorbidities and NSAIDs use among HTN‐C patients (*P*‐value <.0001). NSAIDs use was also significantly higher in older age (*P*‐value <.0001) and female gender (*P*‐value <.02). A high rate of NSAID use was seen among HTN‐C patients with a positive significant correlation to concomitant diseases, older age, and female gender.

**Conclusion:**

The Results of our study indicate that NSAIDs are frequently used among those with HTN‐C and considering the adverse effects of these medication our results further highlight the importance of monitoring and limiting NSAID use.

## 1 INTRODUCTION

Hypertension (HTN), the leading cause of mortality worldwide, was estimated to have affected about 1.39 billion people globally in 2010.^1^ Hypertensive crisis (HTN‐C) is defined as a severe increase in systolic blood pressure, over 180 mmHg, and diastolic blood pressure more than 120 mmHg. Based on whether this crisis causes end‐organ damage or not, it will be classified as emergent or urgent HTN‐C respectively.^2^ Recent studies estimate the prevalence of HTN‐C about 1% to 2% among those suffering from essential HTN.^3^ Non‐steroidal anti‐inflammatory drugs (NSAIDs) are accounted as one of the risk factors for HTN‐C.^4^ NSAIDs are the most common medication prescribed in the medical field which their main use is to treat rheumatological disorders and to reduce pain in general given that they are not addictive and provide pain relief.^5^ They are mainly categorized as selective and non‐selective, while selective NSAIDs mainly inhibit the COX‐2 enzyme, non‐selective NSAIDs inhibit both COX‐1 and COX‐2 enzymes.^6^, ^7^ Despite their beneficial functions, about 30% of hospital admissions regarding preventable harmful drug effects are related to NSAIDs use.^8^ Although different organs may be affected, gastrointestinal problems are the most common adverse events.^9^ In recent years more studies have focused on cardiovascular side effects of NSAIDs and it is now believed that these drugs have great cardiovascular risk and this is especially true in the case of COX‐2 inhibitors or selective NSAIDs.^7^, ^10^ These adverse events include an increased risk of myocardial infarction and HTN.^11^ As a result, when addressing HTN‐C, NSAID use has great importance since uncontrolled HTN plays an important role in developing HTN‐C.^3^ In a recent study, Liew et al reported that among ankylosing spondylitis patients, without baseline HTN, the risk of incident HTN development was 12% higher in those who used NSAIDs continuously compared to negative or noncontinuous use of NSAIDs.^12^ In a cohort study conducted on patients with controlled HTN and negative history of NSAID use, results showed that after 4 years of exposure to NSAIDs more intense HTN treatment will be needed.^13^

The fact that NSAIDs are used commonly and sometimes without prescription prompts us to investigate the extent of safety, potential risks, and adverse effects of these medications. In this study, the aim is to assess the prevalence of NSAID use in patients with HTN‐C and determine whether or not a significant correlation exists between them.

## 2 METHODS

### 2.1 Study design

This is a cross‐sectional study performed between April 2015 and April 2020 in Alzahra hospital, Shiraz. The study was approved by the shiraz university of medical sciences' ethical committee and all patients gave their informed consent before the study in person or through a telephone call.

### 2.2 Participants

The required sample size was estimated to be 257 patients based on previous studies and prevalence of NSAIDs use, with 95% confidence interval. So the number of 257 patients admitted at Alzahra hospital between 2015 and 2020 with a primary diagnosis of HTN‐C who met the inclusion criteria were enrolled in the study. Eligibility criteria included age more than 18 years, clinical diagnosis of HTN‐C, and informed consent. Those who were not willing to participate and patients with incomplete or illegible hospital files and medical records were excluded from the study.

### 2.3 Data collection

Data were collected retrospectively using a researcher‐made questionnaire. In this questionnaire patient's data including demographic and clinical characteristics (name, phone number, age, gender, birth date, history of other illnesses), drug history (NSAIDs, other medications, and hospital drugs), and lab data results (complete blood count [CBC], fasting blood sugar [FBS], blood sugar [BS], blood urea nitrogen [BUN], creatinine [Cr], Na, K) were recorded by assessment of patients' medical records, hospital files and also phone inquiry by a health professional.

### 2.4 Statistical analysis

SPSS 26.0 was used in this study in order to record extracted data and information from questionnaires. Descriptive data were reported as number and percent or mean and SD. The relation between NSAID use and qualitative variables _gender and existence of comorbid diseases in HTN‐C_ was assessed using χ2. T‐test was also administrated to investigate the relation between age and NSAID use among those with HTN‐C. *P* < .05 was considered the limit for statistical significance.

## 3 RESULTS

In our study, information of a total number of 257 patients suffering from HTN‐C meeting eligibility criteria was collected. The mean age of participants was 59.73 years and 62.6% were female. As shown in Table 1 a total number of 137 patients (53.33%) used NSAIDs.

**TABLE 1:** Prevalence of medication use among participants

Variable	Total number	Percent
Aspirin	93	36.1
Non‐aspirin NSAIDs	60	23.2
Both aspirin and non‐aspirin NSAIDs	16	6.2
NSAIDs	137	53.33

Abbreviation: NSAIDs, non‐steroidal anti‐inflammatory drugs.

In the terms of comorbidities, 197 participants (76.7%) had HTN, while 71 (27.6%) and 46 (17.9%) of them suffered from diabetes mellitus (DM) and ischemic heart disease (IHD) respectively. According to Table 2, there was a significant relationship between having HTN, IHD, or DM and NSAID use among those experiencing HTN‐C (*P*‐value <.0001) Also in the case of HTN‐C, a significant relation was found between female gender and NSAIDs use.

**TABLE 2:** NSAIDs use according to comorbid disorder and gender

Variable	Non NSAIDs user	NSAIDs user	*P* value
Negative IHD	117	94	<.0001
Positive IHD	3	43	
Negative DM	103	83	<.0001
Positive DM	17	54	
Negative HTN	48	12	<.0001
Positive HTN	72	125	
Male	57	39	.02
Female	63	98	

Abbreviations: DM, diabetus mellitus; HTN, hypertension; IHD, ischemic heart disease.

Based on statistical analysis, older patients (mean age 64.63 ± 16.04) consumed NSAIDs significantly more than younger (mean age 54.14 ± 15.05) counterparts (*P*‐value <.0001).

## 4 DISCUSSION

Previous studies have already assessed the possible risk factors for HTN‐C^4^, ^14^ but there are few reports regarding the prevalence of NSAID use in a population of patients with HTN‐C specifically. As stated before, our results showed a high rate of NSAID use among HTN‐C patients, also it was concluded that participants use aspirin more than non‐aspirin NSAIDs. Although studies may have associated selective NSAIDs with more cardiovascular risk, this can be explained by the higher rates of aspirin prescription among those suffering from cardiovascular diseases.^15^ Aspirin has been used as primary as well as secondary prevention in atherosclerotic cardiovascular disease (ASCVD) for decades. Yet its role in primary prevention has recently been reevaluated. Based on the 2019 ACC/AHA guideline, aspirin should no longer be used as primary ASCVD prevention in those more than 70 years or those with increased risk of bleeding.^16^ Our results support the limitation of aspirin use as ASCVD primary prevention due to imposed risk of HTN‐C. Also, the high rate of overall NSAID use (53.33%) among participants indicates that our concerns regarding the effects of NSAIDs on HTN‐C must be taken seriously.

We also studied the prevalence of comorbid diseases in HTN‐C and the rate of NSAID use among each of these diseases. Results showed that 76.7%, 27.6%, and 17.9% of participants suffered from HTN, DM, and IHD respectively and NSAID use was significantly higher among all those with comorbidities compared to those who did not have comorbidities. More data are needed to back up these results but this information may help us targeting different patient groups in order to prevent HTN‐C. So, when NSAIDs are required to be administrated in a patient, considering a large number of our participants suffered from comorbid diseases, the safest drug for the existing situation should be considered. Also as previous studies have shown, HTN emergency may occur in patients without a known history of HTN.^17^ As a result, health providers and physicians should be aware of the relation between NSAID use among those with an underlying disorder and therefore prescribe NSAIDs with extra caution and try to limit their use to only when it is necessary to prevent adverse events and HTN‐C.

Age and NSAID use among participants were significantly related in that NSAID use was more prevalent among those with higher age which is in accordance with existing data showing that NSAIDs are mainly used by the elderly.^18^, ^19^, ^20^ Since both blood pressure and HTN tend to increase with age,^1^ and also elderly tend to get more affected by HTN‐C,^3^ higher NSAIDs use in them can be associated with higher rates of HTN‐C. Not to mention that HTN emergencies are also more common in older age.^17^

There are inconsistent data about the prevalence of HTN‐C among different genders.^17^, ^20^ On the other hand, NSAID use is reported to be higher in the female sex.^21^ Wastesson et al also studied paracetamol use among Nordic countries and their investigations showed that this drug is being purchased more by women.^22^ More studies are needed to determine whether higher NSAID use in females may actually lead to a higher incidence of HTN‐C or not.

We conducted this study to investigate whether a relationship exists between NSAID use and HTN‐C and Although our study helps better understanding this relationship, our results cannot be applied in order to deduce a definite correlation between these issues due to study design and sample size. We recommend further cohort studies with larger sample sizes to not only establish the effect of NSAIDs use on HTN‐C but also other factors that may contribute to higher NSAIDs intake among these patients and therefore paving the way for making new guidelines and protocols regarding this matter that will address and inform not only physicians but also patients and community.

## 5 CONCLUSION

Our study results highlight the importance of monitoring NSAID use and especially aspirin in all patients even those who seem to be healthy individuals. These medications are responsible for elevation of blood pressure and therefore HTN treatment intensification which will impose a great burden even if it will not cause HTN‐C. We have to keep in mind that prevention of complications and HTN‐C requires a multidisciplinary approach. NSAIDs are mostly sold over‐the‐counter (OTC) and also prescribed in all medical fields, although adverse effects of them have been studied for some time, their association to HTN‐C still needs more investigation. It is crucial that physicians be aware of the risks and constantly warn their patients especially if they are hypertensive or have its risk factor about the uncontrolled and unsupervised use of NSAIDs and therefore plan regular follow ups to monitor their drug use. Furthermore, based on higher risks of HTN‐C in those who consume aspirin, we suggest that aspirin not only should be avoided as primary ASCVD but also, after initial therapy in those diagnosed with HTN and ASCVD, if medication use is required, monotherapy with clopidogrel or concurrent use of rivaroxaban with aspirin is recommended to lower the risk of HTN‐C development.

## FUNDING

The authors did not receive support from any organization for the submitted work.

## CONFLICT OF INTEREST

All authors certify that they have no affiliations with or involvement in any organization or entity with any financial interest or non‐financial interest in the subject matter or materials discussed in this manuscript.

## AUTHOR CONTRIBUTIONS

Conceptualization: Hamed Bazrafshan.

Investigation: Seyed Hamidreza Mousavi.

Methodology: Hamed Bazrafshan.

Project Administration: Seyed Hamidreza Moosavi.

Supervision: Hamed Bazrafshan.

Writing ‐ Original Draft Preparation: Soodeh Jahangiri.

Writing ‐ Review & Editing: Maryam Salimi, Mohammad Reza Hatamnejad.

All authors have read and approved the final version of the manuscript.

Hamed Bazrafshan had full access to all of the data in this study and takes complete responsibility for the integrity of the data and the accuracy of the data analysis.

## TRANSPARENCY STATEMENT

The lead author (Hamed Bazrafshan) affirms that this manuscript is an honest, accurate, and transparent account of the study being reported; that no important aspects of the study have been omitted; and that any discrepancies from the study as planned (and, if relevant, registered) have been explained.

## CONSENT FOR PUBLICATION

Informed consent was obtained from the patients regarding the publication of this study. There is no identifying information in this article.

## ETHICS STATEMENT

Written Informed consent was obtained from all individual participants included in the study. A copy of the written consent is available for review by the Editor of this journal. The purpose of this research was completely explained to the patient and they were assured that their information will be kept confidential by the researcher. This study was performed in line with the principles of the Declaration of Helsinki. Approval was granted by the ethical committee of Shiraz University of Medical Sciences.

## Data Availability

The authors confirm that the data supporting the findings of this study are available on request from the authors. SPSS data of the participant can be requested from the authors. Please write to the corresponding author if you are interested in such data.
